# Rapid method through routine data to evaluate real-world vaccine effectiveness against coronavirus disease 2019 (COVID-19) infection: lessons from Thailand

**DOI:** 10.1186/s12961-022-00821-6

**Published:** 2022-03-09

**Authors:** Natthaprang Nittayasoot, Panithee Thammawijaya, Piyanit Tharmaphornpilas, Chalo Sansilapin, Chuleeporn Jiraphongsa, Rapeepong Suphanchaimat

**Affiliations:** 1 Department of Disease Control, Ministry of Public Health, Nonthaburi, 11000 Thailand; 2 International Health Policy Program, Ministry of Public Health, Nonthaburi, 11000 Thailand

**Keywords:** Vaccine effectiveness, Coronavirus disease 2019, Case–control, Thailand

## Abstract

The objective of this article is to draw lessons from the Thai experience in estimating vaccine effectiveness (VE) for coronavirus disease 2019 (COVID-19) based on routine service data. We found that a matched case–control design, using probability-based controls representing the varying vaccine coverage across the population over time, yielded a valid result for VE assessment. The proposed design has an advantage in its applicability drawing from the routine data monitoring system. Future studies that exercise other designs, such as test-negative and cohort studies, are recommended in order to compare and contrast the findings across different designs. To implement a continuous monitoring system on VE, the integration of data from different sources is needed. This requires long-term investment in the data monitoring system for the entire healthcare system.

## Background

As the coronavirus disease 2019 (COVID-19) vaccines are massively rolled out in many countries, an increasing number of real-world studies on vaccine effectiveness (VE) are being produced. Most of these studies have utilized an observational study design where the data collected were linked with routine service databases. For instance, Dagan et al. analysed the linked data of the Clalit Health Services in Israel, which covered about 4.7 million records, using a matched cohort study (about 600,000 vaccinated and 600,000 unvaccinated controls) [1]. In the United Kingdom, a test-negative case–control design was conducted. The investigators linked the national vaccine registry data with the reverse transcriptase polymerase chain reaction (RT-PCR) swab results (~ 45,000 cases and 112,000 controls) [2]. Studies in Scotland, Sweden and the United States have exercised a similar study design [2–5]. In Thailand, these data-linked studies are desirable to evaluate the country’s own vaccination effectiveness. However, due to factors including limited experience, the need for multi-organization coordination and privacy law dilemmas, the studies are taking much longer than expected. This delay forced researchers to look for alternative study designs, one of which is presented here.

Thailand’s COVID-19 vaccination programme started in Feb 2021 [6]. At the time of writing (early October 2021), one-dose vaccination coverage in Thailand was at ~ 48% and two-dose coverage amounted to 32% of the population [7]. The majority of vaccines were inactivated vaccines imported from China (CoronaVac) and the domestically produced viral vector vaccine (ChAdOx1). The other vaccines available in Thailand were BBIBP-CorV and Pfizer-BioNTech. CoronaVac was the first vaccine introduced in Thailand. At the time of study (August 2021), the majority of Thai persons had received CoronaVac and ChAdOx1, with about 3,443,840 receiving a second dose of CoronaVac and 903,550 receiving a second dose of ChAdOx1.

All vaccination is required to be reported by both public and private providers to the central immunization database, called the Ministry of Public Health Immunization Center (MOPH-IC). The MOPH-IC system produces a vaccine certificate for a vaccine after each dose. For COVID-19 surveillance, the country has a mandatory surveillance system for reverse transcriptase polymerase chain reaction (RT-PCR)-confirmed COVID-19 cases, as the disease is listed on the roster of dangerous communicable diseases according to the Communicable Diseases Act B.E 2558 (2015) [8]. The Department of Disease Control (DDC) of the MOPH maintains the report. Therefore, compared with other data sources, reliance on the MOPH-IC and the DDC databases ensures minimal chance of encountering missing records.

### Rapid method for evaluating vaccine effectiveness

While the study on the linked national-scale database was ongoing, we attempted to assess VE using the screening method (case–population method) suggested by WHO [8]. As the initial phase of COVID-19 immunization focused on healthcare workers (HCW), and the total number was surveyed prior to vaccine rollout, the daily vaccine coverage could be assumed to be accurate. The source population was set to HCWs in Thailand (including physicians, nurses and laboratory technicians, to name a few, but excluding community health volunteers). To overcome problems of rapidly changing vaccine coverage during accelerated rollout periods, and varied vaccine coverage across provinces due to the distribution policy of the central government, we modified the design into a matched 1:1 case–cohort study with density sampling.

A case was defined as an HCW infected with COVID-19 present in the COVID-19 infectee database. The case immunization history including date of vaccine administration and the name of the vaccine was retrieved from the central immunization database. An effective dose must have been at least 14 days prior to the date of illness (laboratory detected date or date notified if the information on the date of onset was missing). We then created a probability-based matched control using vaccine coverage on the day, using the cumulative vaccine coverage from the beginning of the vaccination campaign until 14 days prior to the date of illness and the residential province of each case.

For example, for VE of two doses versus no vaccine, to match an unvaccinated case that occurred in a province and on the date (−14 days) with 30% of the population unvaccinated, 40% of the population having received one dose of vaccine and 30% having received two doses of vaccine, we first excluded the records with one dose of vaccine only. Then we recalculated the proportion of vaccine coverage for zero doses and two doses of vaccine (this would come up with 50% for zero doses and 50% for two doses). Then, the matched pair would come up with 0.5 concordant pairs and 0.5 discordant pairs. Note that this approach could be applied either for all vaccine types combined or for each vaccine type, as exemplified in the later section where we used CoronaVac as a case study.

Matched data analysis was performed and the results were estimated by McNemar’s chi-square test and presented in the form of odds ratio (OR) and 95% confidence interval (CI). The VE was equal to 1 − OR. Stratified analysis by month or subgroup analysis was performed with the same concept.

### Findings on vaccine effectiveness

**Fig. 1:**
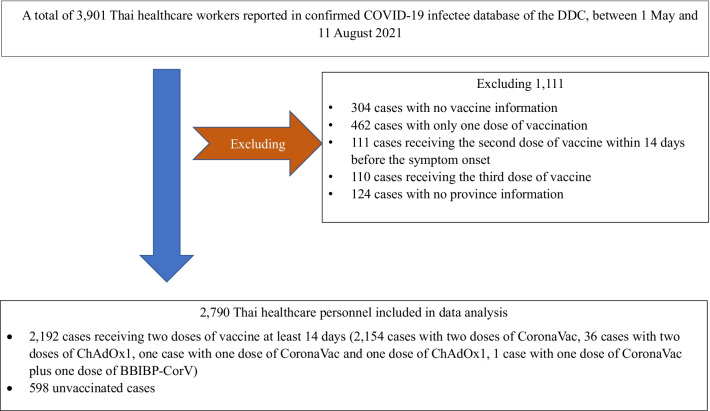
Flow diagram of data used for assessing effectiveness of two doses of CoronaVac, May–June 2021

We estimated the VE of two doses of COVID-19 vaccines in Thailand during May–July 2021 according to the methods described above. Figure 1 presents the flow of the data analysed. Note that people with a single dose or three doses of vaccines were excluded.

Of 4749 healthcare personnel infected with COVID-19 reported between 1 May and 11 August 2021, 3901 were Thai HCW infectees in the DDC database and 2790 were included in the analysis for two-dose VE administration. Most of the participants resided in the epidemic provinces, namely, Bangkok (37.1%), Samut Sakhon (8.7%), Pathum Thani (8.0%) and Nonthaburi Province (7.4%).

Focusing on two doses of CoronaVac, we identified 2752 matched pairs (2154 pairs for cases with two doses of CoronaVac and 598 pairs for cases without vaccine). The McNemar OR is presented in Table 1. The odds of COVID-19 infection amongst participants with two doses of CoronaVac were about a quarter of the odds in the unvaccinated group (OR = 0.28, 95% CI = 0.23–0.33). Therefore, we could conclude that the VE of two doses of CoronaVac against COVID-19 infection was 72% (67–77%). It is worth noting that during May–June, the major SARS-CoV-2 variant in Thailand was B.1.1.7 (Alpha). Then, the fraction of B.1.617.2 (Delta) rose rapidly from approximately 32% at the beginning of July to nearly 80% by the end of the month.

**Table 1:** Matched odds ratio and vaccine effectiveness of CoronaVac against COVID-19 infection, May–June 2021

		Control		Matched odds ratio (95%CI)	Vaccine effectiveness (95%CI)
		CoronaVac (two doses)	No vaccination		
Cases	CoronaVac for at least 14 days	2022	132	0.28 (95% CI 0.23–0.33)	72% (95% CI 67–77%)
	No vaccination	478	120		

**Fig. 2:**
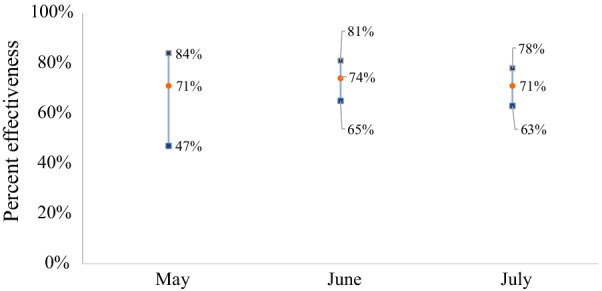
Effectiveness of two doses of CoronaVac by month. Circle = point estimate; square = lower and upper bounds of 95% confidence interval; *p*-value < 0.001 by McNemar’s chi-square test for all months

We found that the results were still robust even after stratification by month. The results indicated that two doses of CoronaVac demonstrated vaccine effectiveness of about 70% in preventing infection (Fig. 2).

Our finding was consistent with the effectiveness of two doses of CoronaVac in Chile (66%) (which mostly responded to the Alpha variant during the time of the study, as in Thailand’s case) but was slightly lower than the findings in other domestic studies, which employed empirical data [9]. For instance, a study in Phuket (one of the famous tourist areas in southern Thailand) showed 91% effectiveness of two doses of CoronaVac against COVID-19 infection in April 2021 [10]. This finding should be interpreted with caution, as the study in Phuket was conducted amongst high-risk contacts in the general population from the local facility’s database, while our analysis focused on HCWs, who tended to face greater risk of infection than other people.

This approach is subject to some limitations. First, compared to conventional case–control studies, it limits the ability to adjust for individual risk of exposure in the calculation, especially for the non-cases for which the vaccination profile was drawn from the entire HCW population. Individual risk always plays an important role in VE analysis. For instance, HCWs who have a higher risk of exposure might be prioritized for vaccination. This means that the confounding risk for the vaccinated could be greater than that for the unvaccinated. Second, the drawing of the percentage of the vaccination status from the source population is just an approximation of the vaccination profile of the non-cases, given that the epidemic has not become extensive. The matched OR (which in turn determines the VE) in this approach is not always exactly the same as the matched OR in the conventional case–control study using true non-cases as a control. However, the probability-based control is conceptually valid, as the drawing of the samples follows a similar approach to the actual drawing of samples from a case-cohort study [11, 12]. The third limitation relates to the concept of matching. We matched a control with each case by residential province and date of illness/diagnosis. Though this approach allows investigators to address any potential confounder originating from the province and time of infection itself, it limits the ability to determine the influence of the province and time on the outcomes. However, this constraint was of minimal concern given that the magnitude of impact of place and time on COVID-19 infection was not the main interest of the study. Moreover, records (either cases or controls) with missing province name and date of illness were excluded. The absence of the province variable did not seriously compromise statistical efficiency because, in our study, only 3.2% of the total records retrieved contained no province information. Lastly, real-world VE study is always subject to the fluctuations of vaccine rollout. Though this issue is more a characteristic of real-world VE study than a limitation in itself, its influence on the validity of this study’s results should not be overlooked.

### Conclusion and the way forward

This study demonstrates the value of simplified analysis in estimating VE through routine vaccine administrative and COVID-19 infectee data. Despite its simplicity, the analysis maintains scientific validity while being feasible for continuous monitoring of VE in situations wherein alternative methods are impractical, or even not allowed. Other countries where VE is of important public health concern may consider applying this approach. However, the Thai Government, particularly the DDC, should invest more in the data recording and analysis system for estimating VE. So far, many real-world studies analysed a short follow-up period. A long-term robust analysis that employs person-time data is likely to be useful. For this purpose, a seamless link between the vaccination database (which includes registered unvaccinated people) and the outcome databases (either the infectee database of the DDC, or the laboratory recording system of local health facilities) is required. Investment in this is not just a matter of monetary support to build up data infrastructure, but also includes strengthening the capacity of local professionals to analyse the data in a timely manner. Also, the VE monitoring results should be regularly communicated to the wider public and should be open to external experts to assess the results’ validity while ensuring confidentiality of data. As questions continue to be asked about the comparative safety of vaccines, the advent of new viral variants, the long-term consequences of COVID-19 and the likelihood of future epidemics, it is crucial to implement these proposals as soon as possible.

## Data Availability

Not applicable.

## Abbreviations

CI: Confidence interval
COVID-19: Coronavirus disease 2019
DDC: Department of Disease Control
HCW: Healthcare worker
MOPH-IC: Ministry of Public Health Immunization Center
OR: Odds ratio
RT-PCR: Reverse transcriptase polymerase chain reaction
VE: Vaccine effectiveness
